# House Cricket (*Acheta domesticus*) and Spirulina (*Arthrospira platensis*) as Non-Conventional Sources of Nutrients and Bioactive Ingredients—Evaluation of Physicochemical, Nutraceutical, and Sensory Properties of Supplemented Muffins

**DOI:** 10.3390/nu17182931

**Published:** 2025-09-11

**Authors:** Ewelina Zielińska, Izabela Podgórska-Kryszczuk, Dawid Ramotowski, Urszula Pankiewicz

**Affiliations:** Department of Analysis and Food Quality Assessment, University of Life Sciences in Lublin, Skromna 8, 20-704 Lublin, Poland; ewelina.zielinska@up.lublin.pl (E.Z.); ramotowskida@gmail.com (D.R.); urszula.pankiewicz@up.lublin.pl (U.P.)

**Keywords:** *Acheta domesticus*, *Arthrospira platensis*, edible insects, microalgae, antioxidant activity, digestion, in vitro predicted glycemic index

## Abstract

**Background:** Non-conventional protein sources, such as edible insects and microalgae, are gaining popularity due to their high nutritional value and environmental benefits. The presented study aimed to examine the effect of a 4% addition of house cricket and spirulina powders on selected properties of muffins. The effects of non-conventional additives on color, textural properties, nutritional value, amino acid composition, and mineral content were determined. **Methods**: Antioxidant activity was evaluated against DPPH· and ABTS·+, and total phenolic content (TPC) and in vitro predicted glycemic index (GI) were examined. Sensory properties were evaluated using a nine-point hedonic scale and a consumer study. **Results**: The results showed that the additions of house cricket and spirulina significantly changed the color parameters of the muffins (ΔE 8.28 and 39.17, respectively) and affected their hardness, cohesiveness, gumminess, and chewiness. Nutritional value was improved, mainly due to an increase in protein content (up to 6% more). Overall, the amino acid profile of the muffins was improved, with a higher presence of all essential amino acids. The enriched muffins had a higher content of selected minerals, including iron, calcium, magnesium, potassium, and zinc, and exhibited higher TPC and antioxidant activity along with a lower in vitro predicted glycemic index. In the consumer evaluation, the spirulina muffins scored highest in texture (8.07 ± 1.04), while cricket muffins received the lowest ratings for color (6.60 ± 1.63), aroma (6.36 ± 2.04), and overall impression (7.03 ± 1.38). Taste did not differ significantly among all muffins. **Conclusions**: The results suggest that muffins can be made using edible insects and microalgae to enhance their nutritional value while maintaining an acceptable taste.

## 1. Introduction

With a growing population, advancing climate change, and pressure to reduce greenhouse gas emissions, modern food systems are forced to seek more efficient and sustainable sources of protein and micronutrients [1]. Unfortunately, animal production contributes significantly to climate change due to high greenhouse gas emissions and progressive deforestation associated with feed cultivation [2]. It is estimated that nearly 10 million hectares are deforested each year, with agricultural expansion responsible for almost 90% of this. Moreover, livestock farming and feed production utilize nearly 80% of all farm land and contribute only 37% of total protein output [3]. At the same time, there has been a growing interest in functional foods that not only provide energy but also offer potential health benefits [4]. In response to these concerns, recent years have seen an increase in interest in the consumption of edible insects (including *Tenebrio molitor*, *Acheta domesticus*, *Locusta migratoria*, *Alphitobius diaperinus* [5]) and microalgae (including *Arthrospira platensis*, *Arthrospira maxima*, *Chlorella vulgaris*, *Odontella aurita*, *Tetraselmis chui*, *Dunaliella salina*, *Haematococcus pluvialis* [6]). They are auspicious ingredients, combining appropriate nutritional value, biological activity, and a lower carbon footprint compared to conventional protein sources [7,8]. Edible insects and microalgae offer a promising alternative strategy for limiting the environmental impact of human development. *Arthrospira platensis*, commonly known as spirulina, holds significant potential and appealing features for large-scale sustainable food production. Spirulina delivers high biomass yields per unit area and can be cultivated on wasteland using non-potable or even saline water [9]. In general, insects are characterized by a lower feed conversion rate (FCR). This rate is defined as the amount of feed required to grow 1 kg of conventional livestock, indicating high feed efficiency. In addition to using less feed and water, insect farming requires less space and emits significantly fewer greenhouse gases than livestock farming [10].

Spirulina and edible insects owe their increasing popularity mainly to their high nutritional value. House crickets (*Acheta domesticus*), for example, have a crude protein content of 63.1% to 76.8% on a dry weight basis and have all the essential amino acids [11,12,13,14]. The species is also characterized by its fatty acid content (e.g., omega-3 and omega-6) and rich micronutrient composition, including potassium, calcium, iron, magnesium, phosphorus, zinc, and manganese [12]. House cricket powder is widely used in food enrichment. Examples of such products include enriched pasta [15], biscuits [16], frankfurters [17], bread [18], muffins [19,20], pancakes [21], and rice noodles [22]. Conversely, the microalga *Arthrospira platensis* is also valued for its rich chemical composition, making it mainly used in the food, pharmaceutical, nutraceutical, and feed industries [23]. Spirulina has “Generally Recognized as Safe” (GRAS) status from the US Food and Drug Administration (FDA), and the World Health Organization (WHO) has called it a “superfood” [24,25]. *Arthrospira platensis* is especially recognized for its high protein content (55–70%), which is highly digestible and contains all essential amino acids. Spirulina is also rich in essential fatty acids, numerous vitamins (including B vitamins, pro-vitamin A, and vitamin E), minerals (mainly potassium, phosphorus, calcium, iron, sodium, and magnesium), and pigments such as chlorophylls and carotenoids [26]. Numerous examples of popular foods enriched with spirulina can be found, including bread [27], broccoli soup [28], basil pesto [29], extruded snacks [30], sourdough crostini [31], gluten-free muffins [32], chocolate milk [33], or kefir [34]. While there are studies on various products containing edible insects and spirulina, none have simultaneously compared the nutritional and health benefits of these two non-conventional protein sources when used in bakery products. Additionally, there is a lack of analysis on consumer perceptions of these enriching ingredients.

Baked goods, including muffins, are a suitable product for fortification, primarily due to their widespread popularity among all social groups, ease of preparation, and affordability. The increasing trend of expanding the variety of functional foods also involves fortification with non-conventional protein sources that can benefit the human body. A particular limitation in the introduction of new, previously unfamiliar foods is the phenomenon of food neophobia [35]. This barrier is evident not only in the case of foods with added insects in areas where entomophagy has only recently been introduced but also in areas where insects are traditionally consumed but their consumption is declining due to changing eating habits [36]. Another limitation to using non-conventional protein sources is the low consumer awareness of the rich nutritional content of microalgae and edible insects as well as their potential health and environmental benefits. Therefore, ongoing efforts are necessary to raise consumer awareness of non-conventional protein sources, such as spirulina and edible insects. Consumer evaluation of designed products is a crucial aspect of research on non-conventional protein sources. The acceptance of new products by consumers is affected by various characteristics, including color, smell, texture, and taste. Additionally, consumers’ beliefs, emotions, and associations with non-conventional ingredients also play a significant role in their acceptance. A strong example of this correlation is that individuals who possess positive associations with insects or have more knowledge about their benefits exhibit a greater willingness to try products containing them [37]. Moreover, research has highlighted differences between individuals who have tried edible insects at least once and those who have not. This can be attributed to the idea that taste is a key factor influencing food preferences. Positive taste experiences can enhance familiarity with new ingredients derived from non-conventional food [38]. Sogari et al. propose that individuals who have previously tried eating insects are more likely to consume and purchase them again compared to those who have never had the opportunity [36]. This consumer research serves a dual purpose: it not only encourages respondents and their family members to consider consuming these products by highlighting positive reactions but also plays a crucial role in the application of the developed recipes within the food market.

The purpose of the study was to examine selected properties of muffins, a popular confectionery product, enriched with a 4% addition of house cricket (*Acheta domesticus*) and spirulina (*Arthrospira platensis*) powders. Based on our previous research, we chose a level of enrichment that achieves the desired nutritional and health benefits while ensuring full consumer acceptance [20,26,29,39]. The study included evaluation of physical, nutritional, bioactive, and sensory properties. The presented manuscript provides valuable dietary information, and the newly developed products can be highly competitive in the confectionery market due to their beneficial properties. Furthermore, the consumer survey conducted provides valuable insights for food manufacturers.

## 2. Materials and Methods

### 2.1. Materials

Wheat flour (type 405) (protein 10%, fat 1%, carbohydrates 71.7%) (GoodMills Polska sp. z o.o., Grodzisk Wielkopolski, Poland), sugar (Pfeifer & Langen Polska S.A., Środa Wielkopolska, Poland), rapeseed oil (Bunge Polska Sp. z o.o., Kruszwica, Poland), milk (Okręgowa Spółdzielnia Mleczarska, Łowicz, Poland), eggs (OVOS sp. z o.o., Bieżuń, Poland), baking powder (FoodCare sp. z o.o., Zabierzów, Poland), and spirulina powder from organic cultivation (Medicaline Konrad Malitka, Karczew, Poland) were purchased from the local supermarket. The crickets *Acheta domesticus* (Fabricius, Orthoptera: Gryllidae) (adult) were obtained from a commercial supplier from Poland (CricketsFarm, Lublin, Poland). All chemicals and reagents used were of analytical grade.

### 2.2. Production of Insect Powder

Crickets were lyophilized for approximately 48 h to achieve a moisture content of less than 10%. Subsequently, they were ground in a laboratory mill to produce particles smaller than 850 µm, and to ensure proper particle size control, they were sifted through a 20-mesh sieve. The powder was vacuum-packed and stored in a frozen state until needed.

### 2.3. Muffin Preparation

Muffins were made in two variations: using spirulina or house cricket powder in place of wheat flour at a 4% substitution rate. The control muffins were made without adding any of the test raw materials. The muffin recipe included common ingredients: wheat flour (31.6%), milk (29.2%), eggs (14.0%), rapeseed oil (12.3%), sugar (11.7%), and baking powder (1.2%). The wet and dry ingredients were combined separately and then briefly mixed to combine. The dough (57 g) was transferred to muffin molds and baked in an oven preheated to 180 °C for 20 min. Once baked, the muffins were allowed to cool to room temperature before being prepared for further analysis.

### 2.4. Physical Analysis

#### 2.4.1. Color Measurements

The EnviSense NH310 colorimeter (EnviSense, Lublin, Poland) was used to measure the color of the muffins. The color difference was measured in the CIE L*a*b* color space. The color parameters were described by L*—brightness (0—black, 100—white), a* (−a*—green, +a*—red), and b* (−b*—blue, +b*—yellow). Additionally, chroma (C*) and hue (h°) were also measured. The total color difference was calculated using the following formula [20]:

$$∆E=\sqrt{{∆L}^{2}+{∆a}^{2}+{∆b}^{2}}$$

where ΔL*, Δa*, and Δb* are differences in the L*, a*, and b* values, respectively, between the reference sample and the test sample.

The whiteness index (WI) was calculated using the measured L*, a*, and b* values as follows [40]:

$$WI=100-\sqrt{{\left(100-L^{*}\right)}^{2}+{a^{*}}^{2}+{b^{*}}^{2}}$$

#### 2.4.2. Texture Profile Analysis (TPA)

Muffin crumb texture analysis was conducted the day after baking using a texturometer (BROOKFIELD PRO CT3, Scarsdale, NY, USA) equipped with a 25 kg load cell and a 10 cm × 15 cm metal plate probe. The muffins were cut into pieces measuring 3 cm × 3 cm × 2 cm and pressed twice to 50% of their original height. Measurements were taken at a head travel speed of 2 mm/s, with an interval of 5 s between sampler movements. The following parameters were evaluated: hardness (g), cohesiveness, springiness (mm), gumminess (g), chewiness (mJ). The results were recorded in the TexturePro CT V1.6 computer software.

### 2.5. Nutrient Composition

Using standard analytical methods, muffin, insect, and spirulina powders were assessed for moisture, ash, fat, and protein content (N × 6.25 or 5.09 for cricket powder [41]) [42]. Moisture content was measured by drying the samples at 120 °C in an oven (SUP-65W, Wamed, Warsaw, Poland) until a constant weight was achieved. The ash content was determined by ashing the samples and then heating the residue to a constant mass at 500 °C in a muffle furnace (Czylok, Jastrzębie Zdrój, Poland). Crude fat was determined using the Soxhlet extraction method (Tecator Soxtec, type HT 1043, Gemini, Apeldoorn, Sweden) with hexane as the extractant. The crude protein content (N × 6.25) was analyzed using the Kjeldahl method in a Kjeltec 8400 automatic distiller (Foss, Foss Analytical AB, Höganäs, Sweden). The carbohydrate content was determined using the formula 100 − [weight in grams (moisture + protein + fat + ash) in 100 g of fresh muffins]. The nutritional value was calculated using the conversion method, with conversion factors of 9 × (g fat) and 4 × (g protein + g carbohydrate) [43].

### 2.6. Determination of the Amino Acid Composition, Calculation of the Limiting Amino Acid Index, and the Essential Amino Acid Integrated Index (EAAI)

The amino acid content of the samples was determined using the AAA-400 automatic amino acid analyzer (Ingos Ltd., Prague, Czech Republic), and the test was performed at the Central Research Laboratory of the University of Life Sciences in Lublin.

Protein quality was assessed based on the amino acid score, which is a calculation comparing the amount of an essential amino acid in the protein with the amount of that amino acid in a reference protein:

$$Amino\;acid\;score=\frac{mg\;of\;amino\;acid\;in\;1\;g\;of\;the\;test\;protein}{mg\;of\;amino\;acid\;in\;1\;g\;of\;reference\;protein}$$

An essential amino acid index (EAAI) was also calculated based on the content of all essential amino acids compared to a reference protein, specifically the value for human needs [44].

### 2.7. Minerals Content

To 0.5 g of each lyophilized sample, 4 mL of nitric acid (V) was added, and then the mixture was mineralized in a microwave oven at 200 °C for 20 min (Mars, Xpress, CEM Corporation, Matthews, NC, USA). The resulting mineralizates were transferred to 25 mL volumetric flasks and filled with deionized water to the mark. The concentration of mineral ions in the mineralizates was determined by flame atomic absorption spectrophotometry (FAAS, Solaar 939, Unicam, Ilminster, UK) using an acetylene-air flame at 766.5 nm for potassium, 589 nm for sodium, 422.7 nm for calcium, 213.9 nm for zinc, 248.3 nm for iron, and 285.2 nm for magnesium [45].

### 2.8. Antioxidant Properties

#### 2.8.1. Extraction of Bioactive Compounds

Muffin samples (1 g each) were homogenized using a laboratory grinder and extracted with 10 mL of an ethanol-water mixture (4:1, *v*/*v*). The samples were shaken for 120 min in a laboratory shaker. After shaking, the mixtures were centrifuged at 3000× *g* for 10 min. The resulting supernatants were then stored at −18 °C until further analysis the following day [46].

#### 2.8.2. DPPH Radical Scavenging Activity

The DPPH assay was conducted following the method described by Brand-Williams et al. [47], with minor modifications. A 0.1 mL portion of the sample was mixed with 0.9 mL of a 6 μM DPPH· solution prepared in 75% methanol. After allowing the reaction to proceed for 30 min, the absorbance was measured at 515 nm, using 75% methanol as the blank. The scavenging activity was calculated using the following formula:

Scavenging activity (%) = [1 − (A sample/A control)] × 100

where A sample—absorbance of the mixture of sample and DPPH·, and A control—absorbance of the DPPH· solution alone.

Results were expressed as Trolox Equivalent Antioxidant Capacity (TEAC) in mM Trolox.

#### 2.8.3. ABTS Radical Scavenging Activity

The ABTS assay was conducted following the protocol established by Re et al. [48], with minor adjustments to the volumes of antioxidants used. In this method, 2.9 mL of the ABTS·^+^ solution was mixed with 0.1 mL of the sample. After allowing the mixture to stand for 30 min, the absorbance was measured at 734 nm using deionized water as the blank. The scavenging capacity was then calculated as follows:

Scavenging activity (%) = [1 − (A sample/A control)] × 100

where A sample—absorbance of the mixture of sample and ABTS·^+^, and A control—absorbance of the ABTS·^+^ solution alone.

Results were presented as TEAC values (mM Trolox).

#### 2.8.4. Total Phenolic Content (TPC)

The total phenolic content was evaluated using the Folin–Ciocalteu spectrophotometric method as described by Singleton and Rossi [49]. In brief, 0.2 mL of the extract was combined with 3 mL of distilled water and 0.2 mL of Folin–Ciocalteu reagent. After vortexing the mixture, 0.6 mL of saturated sodium carbonate was added, and the solution was incubated at 40 °C for 30 min. The absorbance was measured at 765 nm. The results were expressed as milligrams of gallic acid equivalents (GAE) per 100 g of sample.

### 2.9. Predicted Glycemic Index In Vitro

The predicted glycemic index (GI) of muffin samples was determined in vitro by evaluating their starch digestibility using a modified method from Monro et al. [50]. The digestion process was carried out in a dark environment at 37 °C with stirring at 130 rpm. A total of 30 mL of water and 0.8 mL of 1 M HCl were added to 1 g of the sample to achieve a pH of 2.5. Gastric digestion was initiated by adding 1 mL of a 10% pepsin solution in 0.05 M HCl. Following 30 min of digestion, the small intestine phase began. For this phase, 2 mL of 1 M NaHCO_3_ and 5 mL of a 0.1 M phosphate buffer (pH 6) were added, followed by the addition of 4.6 mg of amyloglucosidase and 5 mL of 2.5% pancreatin in 0.1 M of phosphate buffer (pH 6). The total volume of hydrolysates was adjusted to 55 mL using distilled water. According to the Reis and Abu-Ghannam method [51], at intervals of 0, 20, 30, 60, 90, 120, and 180 min after the start of amylolysis, 1.0 mL aliquots of digesta were transferred to 4 mL of absolute ethanol to inactivate the enzymes. The GOPOD (Glucose Oxidase Peroxidase) method was used to determine the glucose content of the samples (mg glucose/g sample). The glucose content was then plotted as a function of time, and the areas under the hydrolysis curves (AUCs) were calculated. The hydrolysis index (HI) was determined by calculating the ratio of the AUC of the sample to the AUC of the reference food, which was white bread. The in vitro predicted glycemic index was calculated using the formula established by Goñi, Garcia-Alonso, and Saura-Calixto [52]:

GI (%) = 39.71 + 0.549 × HI

### 2.10. Consumer Acceptance Evaluation

The muffins were divided into portions, each assigned a three-digit code, and were evaluated by a group of 70 participants (47 women and 23 men) aged between 19 and 36 years. The participants in the study were students and employees of the University of Life Sciences in Lublin. Consumers participating in the survey received Information for Study Participants and completed an Informed Consent Form for Participation in the Study. Two methods were employed to assess the participants’ acceptance of the muffins. Preference was measured using a 9-point hedonic scale, with individual ratings explained as follows: 1 indicated “dislike extremely,” 5 represented “neither like nor dislike,” and 9 signified “like extremely” [53]. The second evaluation method was the ranking method, in which consumers rated the samples, assigning them ranks from best (1) to worst (3). Points were awarded based on the rankings: 3 points for 1st place, 2 points for 2nd place, and 1 point for 3rd place. Participants rinsed their mouths with plain water before each trial.

### 2.11. Consumer Study

The consumer study was conducted in two stages: first, before the organoleptic evaluation of the muffins, and then after. Participants responded to a series of statements using a 5-point Likert scale, with options ranging from “strongly disagree” to “strongly agree” [54]. The objective of the study was to capture consumers’ perceptions of the muffins both before and after tasting. Additionally, the study aimed to assess their willingness to consume insects in the future, taking into account their sensory experiences. The statements presented prior to the sensory evaluation included:

1. I know the benefits of consuming non-conventional protein sources (e.g., insects, microalgae).
2. I am curious about the taste of muffins that have had spirulina and insect flour added.
3. All muffin variants look equally appetizing.
4. The non-conventional additives made me reluctant to try the muffins.

In addition, the consumer study card included a statement regarding previous consumption of products with non-conventional protein sources, to which the consumer could answer “yes” or “no”.

Following the sensory evaluation of the muffins, participants were requested to evaluate the following aspects:

1. The muffins with non-conventional additives positively surprised me in terms of taste.
2. Muffins with non-conventional protein sources are acceptable to me, and I would eat them in the future.
3. I would be interested in trying other baked goods with insect flour or spirulina.
4. I would recommend trying baked goods made with insect flour or spirulina to others.

### 2.12. Statistical Analysis

All experiments were conducted with a minimum of three replicates. The results are presented as mean values accompanied by their standard deviations. Statistical analysis was carried out using Statistica 13.3 (Statsoft, Krakow, Poland) and Excel 2019 (Microsoft, Washington, DC, USA). The validity of the collected data was confirmed to have a normal distribution using the Shapiro–Wilk test. Since the data conformed to a normal distribution, we conducted the parametric statistical analysis using a one-way ANOVA. Because the ANOVA was statistically significant, we followed up with the Tukey post hoc test. All statistical hypotheses were assessed at a significance level of *p* < 0.05.

## 3. Results and Discussion

### 3.1. Color Measurements

**Figure 1:**
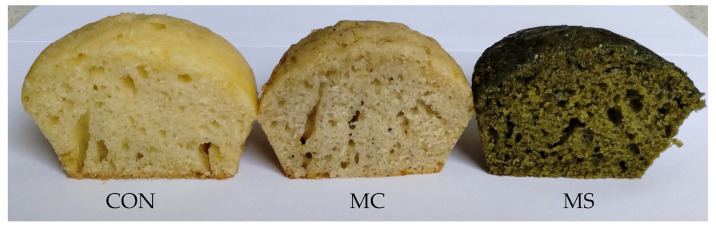
Appearance of the obtained muffins. CON—control sample; MC—muffin with cricket; MS—muffin with spirulina.

Consumer acceptance of food relies on factors beyond its composition, recipe, or taste; a significant aspect of consumer perception is the product’s color. The results of the color measurements are presented in Table 1, and the general appearance of the prepared muffins is shown in Figure 1. The addition of non-conventional protein sources to the muffins significantly altered their color characteristics. For muffins with cricket powder, the lightness (L* 65.08 ± 0.81) and yellowness (b* 20.13 ± 0.42) values decreased compared to the control, while the redness (a* 3.41 ± 0.25) value increased, indicating a shift toward a more reddish color. Conversely, for baked goods containing spirulina, the L* value also decreased (32.62 ± 1.06), but the a* (7.78 ± 0.71) and b* (30.43 ± 0.71) values increased, indicating a shift towards more red and yellow hues. This color change can be attributed to the thermal decomposition of pigments found in spirulina, particularly phycocyanin, which is sensitive to high temperatures (above 50 °C). High temperatures cause alterations in chromophore stability and color changes, which are probably linked to changes in the secondary, tertiary, and quaternary structures of the protein. Phycocyanin, a blue-green protein pigment that imparts spirulina its intense color, has found use in the food industry. Interest in phycocyanin is increasing steadily due to its antioxidant, anticancer, and anti-inflammatory qualities. However, as already mentioned, this dye is characterized by low stability during storage and food processing due to its sensitivity to high temperatures as well as the presence of light and extreme pH values [55].

Other researchers have observed changes in the color of the resulting products based on the type of additive used. For instance, insect powder and spirulina exhibit a darker color compared to wheat flour, which directly influences the appearance of the baked goods. Consistent with previous findings, the addition of cricket powder to cakes, as reported by Bawa et al. [56], significantly altered the color by decreasing the L*, b*, and C* values. Similarly, muffins are affected by the inclusion of other microalgae, such as *Chlorella vulgaris*. A study by Marzec et al. [57] on vegan muffins with this microalgae also revealed a significant change in the color parameters of the baked goods after incorporating the investigated material.

The addition of non-conventional protein sources tested increased total color difference (ΔE). The total color difference was measured at 8.28 for the muffins made with insect flour and 39.17 for those made with spirulina. Both values were greater than 3, indicating that the differences were noticeable to the human eye [58]. For muffins made with insect flour, the color saturation was lower, as reflected by a lower C* value (20.33 ± 0.47). In contrast, muffins containing spirulina exhibited a higher C* value (31.41 ± 0.60), indicating increased color saturation. The color of the product depends on several factors, including the color of the ingredients used—insect powder and spirulina are darker than wheat flour. The observed color changes may also be attributed to the conversion of nutrients found in the muffins, such as proteins from insects and spirulina, during the heat treatment applied. The enriched muffins had a higher protein content, which may have intensified the Maillard reactions [59].

### 3.2. Instrumental Texture Profile Analysis

Texture, in addition to taste perception, is one of the most important distinguishing features of food quality. Muffins, as sponge-fat baked goods, should feature a soft and delicate crumb, reflecting low hardness and chewiness while maintaining high elasticity [57]. The addition of non-conventional protein sources, such as cricket powder and spirulina, significantly affected changes in specific texture parameters. The results of the texture measurements are shown in Table 2. The most significant changes compared to the control sample were observed in the case of baked goods with added spirulina. The hardness parameter decreased from a value of 2754.33 ± 90.64 g to 1434.00 ± 80.73 g—a decrease of more than 52%. In the case of muffins with the addition of cricket powder, the hardness decreased by almost 32%. Research has shown that substituting wheat flour with gluten-free flours, such as rice, quinoa, and millet, in muffin recipes leads to a decrease in hardness [60,61]. Kowalczewski et al. [62] reported that the hardness decreased as the amount of cricket powder increased in the gluten-free bread formula. They suggested that the softening effect of cricket powder may be linked to the emulsifying properties of cricket proteins, which are known for their excellent emulsifying capabilities [63] because similar effects on the structure of crumbs were previously observed after adding natural emulsifiers to dough [62]. According to Feili et al. [64], the hardness of baked products mainly depends on the amylopectin and amylose content of the product. The water absorption capacity of insects and spirulina powders can be quite important. However, some studies indicate that a moisture content difference of around 5% in baked goods does not significantly impact the texture parameters [20]. Moreover, the addition of the tested raw materials affected the cohesiveness of the baked goods obtained. It increased from 0.51 ± 0.01 to 0.56 ± 0.02 for both samples with non-conventional protein. The parameter that did not change significantly after the addition of non-conventional protein sources to the muffins was springiness. Springiness refers to how well a muffin returns to its original form after being compressed. It serves as an indicator of freshness, with higher springiness suggesting a better-quality product. Lower springiness suggests unfavorable changes in consumer acceptance, such as a tendency for the product to crumble. Additionally, reduced springiness and cohesiveness point to a lower specific volume and a less aerated muffin structure [65]. As with hardness, the values of the gumminess and chewiness parameters were highest for the control sample and lowest in the muffins with spirulina (gumminess of 1416.00 ± 21.00 g for the control and 861.00 ± 49.52 g for the muffins with spirulina and chewiness of 144.03 ± 10.01 mJ and 77.20 ± 2.63 mJ, respectively). A lower chewiness means it takes less time to chew a muffin before swallowing [66].

Other researchers have also observed changes in the texture parameters of products with the addition of edible insects or spirulina. A study by Pauter et al. [59] demonstrated a decrease in hardness, elasticity, and chewiness in muffins that contained cricket powder. A study by Zielińska et al. [20] found that supplementing muffins with insect powder resulted in a softer texture compared to the control group. This was demonstrated by a significant decrease (*p* < 0.05) in hardness, springiness, resilience, cohesiveness, and chewiness of the muffins. Additionally, the inclusion of cricket powder (*Gryllodes sigillatus*) had a more pronounced impact on the texture of the muffins than mealworm powder (*Tenebrio molitor*). Djouadi et al. [67] also noted alterations in the texture parameters of crackers made with *Tenebrio molitor*. With an increased addition of insect powder, there was a corresponding decrease in hardness and a reduction in the time required to cause cracking, indicating that crackers with insect content were softer. Additionally, studies conducted by Moradi et al. [68] on bread, cake, and layered sweets, as well as by Sanjari et al. [69] on baguettes, found that the inclusion of spirulina also reduced the hardness of the tested products, alongside significant decreases in various texture scores, including stiffness, chewing ability, porosity, and elasticity.

### 3.3. Nutrient Composition

The nutritional value of the tested muffin samples and the *Acheta domesticus* and *Arthrospira platensis* used to enrich are shown in Table 3. Edible insects and spirulina are both valuable sources of protein, as proven in the study presented here. The protein content of the powder from *Acheta domesticus* was 73.24 ± 0.17 g/100 g FM (fresh material), and that of *Arthrospira platensis* was 70.20 ± 0.23 g/100 g FM. Consequently, enhancing the muffins with cricket powder and spirulina led to a notable increase in the protein content of the newly produced product, recorded at 7.19 ± 0.06 g/100 g FM and 7.10 ± 0.05 g/100 g FM, respectively. Powder from *Acheta domesticus* contained a higher fat content (12.01 ± 0.04 g/100 g FM) than *Arthrospira platensis* (5.60 ± 0.01 g/100 g FM), resulting in a significant increase in fat content in muffins with edible insects (15.54 ± 0.11 g/100 g FM). Due to the low carbohydrate content of cricket powder (0.8 ± 0.08 g/100 g FM), muffins fortified with this additive had a significantly lower carbohydrate content (38.47 ± 0.77 g/100 g FM) compared to controls (40.22 ± 0.4 g/100 g FM) or products with spirulina (39.91 ± 0.4 g/100 g FM). Spirulina had a higher ash content (6.21 ± 0.03 g/100 g FM) compared to cricket powder (4.43 ± 0.03 g/100 g FM). Thereby, the microalgae-enriched muffins had significantly higher ash content (1.28 ± 0.04 g/100 g FM) than the control muffins (1.19 ± 0.01 g/100 g FM) or those enriched with insect powder (1.21 ± 0.02 g/100 g FM). Regarding water content, neither the control muffins nor the two enriched variants showed significant differences from one another. Muffins with insect powder had the highest energy value (322.50 kcal/100 g), primarily due to their significantly higher fat content.

The use of edible insects and microalgae for fortification in popular food products appears suitable for enhancing specific nutritional parameters, primarily protein. Spirulina contains significantly higher amounts of protein (55–70%) compared to commonly consumed foods [26]. Furthermore, scientific studies show that spirulina protein is highly digestible (around 85% on average) [70], mainly due to the structure of the cell wall of *Arthrospira platensis*, which is primarily made up of proteins, carbohydrates, and lipids [71]. This is particularly important for vegans and vegetarians because, in contrast, the cell wall of plants is made of cellulose that is indigestible to humans [72]. The protein content of most edible insects ranges from 13% to 80% and depends on many factors, e.g., their metamorphosis stages, sex, feeding mixtures used, and varying water content. Some insects are rich in fat, with a wide range of fat content, from 4.56% to 60%, and a high content of unsaturated fatty acids. The fat content is higher in the larval stage than in the adult stage. The low carbohydrate content is mainly due to chitin [10]. In general, the nutritional values of edible insects vary significantly. It is complex to generalize about the nutritional value of the approximately 2000 edible insect species; however, insects serve as an excellent food enrichment ingredient, much like spirulina.

### 3.4. Amino Acid Composition and the Chemical Score of Protein Quality

The amino acid composition of a protein is crucial in assessing its quality. A high-quality protein presents a complete and optimal essential amino acid composition for human needs. It is crucial to recognize that both powders studied are sources of complete protein (Table 4). The sum of essential amino acids in spirulina powder was 312.2 mg/g of protein, and in cricket powder, it was 294 mg/g of protein, which was higher than the essential amino acid content in the reference protein [73]. The total amino acid content was 709.4 mg/g of protein and 711.3 mg/g of protein, respectively. The use of the tested powders as an enrichment for the muffins increased their amino acid content relative to the control. All amino acids, except glutamic acid and proline, were present at higher levels in the muffin with spirulina than in the control (*p* < 0.05). Generally, the sum of essential amino acids in a muffin with spirulina was 40.25 mg/g protein and in a muffin with cricket was 38.71 mg/g protein, compared to 36.6 mg/g protein for the control. An increase in total amino acids is also noticeable—98.55 mg/g protein for the muffin with cricket and 101.92 mg/g protein for the muffin with spirulina compared to 95.18 mg/g protein for the control. Among the essential amino acids, lysine, methionine, cysteine, and threonine exhibited the highest average increases in muffins fortified with cricket and spirulina powders, containing 15.2% and 16.9%, 9.8% and 14.8%, 10.4% and 12.9%, 7.6% and 13.3%, respectively. Among the non-essential amino acids, alanine showed the highest increase, with a 27% rise in muffins fortified with cricket powder and a 24.5% rise in muffins containing spirulina powder.

In this study, two important chemical parameters, AAS and EAAI, were used to assess protein quality. Protein quality can be evaluated using a chemical score (CS), which compares the essential amino acid content of a test sample to that of a reference protein. As tyrosine can substitute phenylalanine, through metabolic processes, these amino acids were combined (tyrosine + phenylalanine) for the calculation of AAS [74]. The essential amino acid index (EAAI) evaluates the content of all essential amino acids in relation to a reference protein, which is selected based on human nutritional requirements. The amino acid with the lowest CS is the limiting amino acid in the protein being tested [44]. EAAI reflects more the biological quality of a protein than AAS. The limiting amino acid in the muffins is lysine (Table 5). Chemical score for lysine showed the highest increase in the supplemented muffins compared to the control (increased by 16.8% for muffin with spirulina powder and 15.2% for muffin with cricket powder). In cereal products, lysine is a limiting amino acid; therefore, the enrichment process enabled it to be utilized to a greater extent. The calculated essential amino acid index (EAAI) of supplemented muffins with cricket (18.38) and with spirulina (19.04) is higher than that of the control (17.12). A high EAAI value generally characterizes a protein with high quality and efficiency. Brown and Jeffrey [75] state that a protein is considered high quality when its EAAI value exceeds 90%. It is regarded as moderate quality when the EAAI falls between 70% and 89%, and low quality when the EAAI is below 70%. Based on the reference standard used, the proteins of the analyzed insects and spirulina can be considered of high quality, with EAAI values of 112.84% and 123.09%, respectively.

### 3.5. Mineral Content

The mineral content of the muffins and the powders used to enrich them is shown in Table 6. Enriching muffins with *Arthrospira platensis* powder resulted in a significant increase in selected elements in the product, including calcium (48.83 ± 0.29 mg/100 g FM), magnesium (16.30 ± 0.05 mg/100 g FM), potassium (126.72 ± 0.32 mg/100 g FM), sodium (251.17 ± 0.81 mg/100 g FM), and iron (1.50 ± 0.05 mg/100 g FM). Special attention should be given to iron, which is present in high amounts in the tested spirulina powder (35.59 ± 0.20 mg/100 g FM), resulting in the fortified muffins containing as much as twice the iron content of the control. Furthermore, both *Acheta domesticus* and *Arthrospira platensis* are rich sources of potassium, which was detected in the tested powders at 1296.22 ± 3.03 mg/100 g FM and 1448.41 ± 1.21 mg/100 g FM, respectively. Muffins enriched with cricket powder had increased calcium (48.05 ± 0.15 mg/100 g FM), potassium (126.65 ± 0.34 mg/100 g FM), and zinc (0.78 ± 0.02 mg/100 g FM).

Edible insects and microalgae serve as valuable sources of selected minerals, as shown in the study presented here. Replacing a small portion of flour (4%) with *Acheta domesticus* and *Arthrospira platensis* powders in popular, easy-to-prepare baked goods yields muffins rich in essential elements necessary for the proper functioning of the body. Notably, the increase in iron content in spirulina-enriched muffins is significant, given that iron deficiency is one of the most common nutritional issues worldwide [76]. Vegans and vegetarians in particular are at risk of iron deficiency, which is associated with reduced absorption of non-heme iron. In contrast, iron from spirulina is mostly bioavailable [77]. Also noteworthy is the fact that the magnesium content in muffins fortified with *Arthrospira platensis* powder increased by more than 40%. This element plays a crucial role in many physiological functions. Magnesium deficiency causes changes in biochemical pathways, which increase the risk of chronic degenerative diseases [78]. Notably, the muffins enriched with cricket powder also contained more than 50% more zinc compared to the non-enriched muffins. This element is essential for normal growth and development. Zinc also plays a role in the formation of protein structures and enzymes. Zinc-regulated cell signaling pathways are involved in numerous biological functions, including cell growth and proliferation, apoptosis, immune response, and the synthesis and secretion of certain hormones [79]. Importantly, from a nutritional perspective, the study revealed over a 10% increase in calcium content in muffins enriched with cricket powder and microalgae. This element is not only a fundamental component of bones, teeth, and connective tissue but also plays a role in many vital functions, including nerve impulse transmission, muscle contraction, blood clotting, enzyme activation, immune response, and cell differentiation [80]. Enriching the muffins with powder from *Acheta domesticus* and *Arthrospira platensis* also resulted in a near 16% increase in potassium content. Potassium affects many processes in the human body, including being a cofactor involved in protein synthesis, enzyme activation, and water management. The element is necessary for carbohydrate metabolism, insulin secretion, or creatine phosphorylation. Diets rich in potassium are associated with a reduction in blood pressure, thereby reducing the risk of cardiovascular disease or stroke [81].

### 3.6. Antioxidant Properties

The antioxidant properties of muffins containing house cricket and spirulina powders were evaluated by measuring the total polyphenol content (TPC) and assessing their antioxidant activity against the ABTS·+ and DPPH· assays. The results of these tests are presented in Table 7. The lowest TPC was recorded for muffins without functional additives (18.42 mg GAE/100 g) and the highest for muffins with the addition of house cricket (26.47 mg GAE/100 g). The result of a significant increase (more than 40%) in polyphenol content is similar to that obtained by Ivanišova et al. [82], who studied crackers supplemented with 5% powders from edible insects. They found that the control crackers had a phenolic compound content of 0.49 mg GAE/g, whereas crackers supplemented with cricket powder had a content of 0.71 mg GAE/g. In the study presented here, the TPC value for spirulina-enriched muffins was 20.20 mg GAE/100 g, which was statistically significantly higher than the control. The addition of spirulina to food products also increases TPC, as indicated by other authors’ results [31,83,84]. However, the level of polyphenol growth varies, and these differences may be due to the characteristics of the samples tested, the method of microalgae cultivation, geographical origin, and even environmental changes [84].

Similar results were also obtained for the ability to neutralize DPPH· and ABTS·+ by muffin extracts. The control muffins showed the lowest antiradical activity (DPPH· scavenging activity 0.018 ± 0.002 mM TE/100 g, *p* < 0.05 and for ABTS·+ 0.126 ± 0.001 mM TE/100 g, *p* < 0.05), and the muffins with cricket powder added had the highest antioxidant activity (DPPH· scavenging activity 0.087 ± 0.003 mM TE/100 g, *p* < 0.05 and for ABTS·+ 0.211 ± 0.005 mM TE/100 g, *p* < 0.05, respectively). The results obtained are consistent with those of the previously mentioned studies on crackers [82]. There, the value of antioxidant activity against DPPH· increased from 1.93 mg TEAC/g for the control sample to 2.56 mg TEAC/g for cricket crackers. An increase in antioxidant activity against ABTS·+ and DPPH· was also noted for sponge cakes with the addition of flours from edible insects [85]. In the case of standard sponge cake, ABTS·+ and DPPH· values were 5.98 mg Tx/g and 4.95 mg Tx/g, respectively. For sponge cake with 30% cricket flour, the ABTS·+ scavenging activity was 15.93 mg Tx/g, and for DPPH· it was 13.2 mg Tx/g. Spirulina-enriched muffins also exhibited increased antioxidant activity, with values of 0.053 mM TE/100 g for DPPH· and 0.163 mM TE/100 g for ABTS·+. Other authors also confirm that the addition of spirulina to various foods increases antioxidant activity. Antioxidant activity values against ABTS·+ for spirulina-enriched vegan cakes [86] ranged from 6.41 to 21.31 μmol TE/100 g. In another study on gluten-free spirulina-enriched crackers [87], spirulina addition significantly increased TPC, DPPH·, and ABTS·+ values.

The results of our studies and those of the cited authors indicate that both edible insects and spirulina exhibit possible potent antioxidant activity. Further studies are recommended to confirm these properties in vivo. The antioxidant activity of microalgae is a result of their abundance of compounds that can scavenge free radicals, including phenolic compounds, carotenoids, or flavonoids and the blue pigment phycocyanin found in spirulina [88]. *S. platensis* is considered an excellent source of phenolic acids, including caffeic acid, chlorogenic acid, salicylic acid, synaptic acid, and trans-cinnamic acid [89]. The phycocyanin and its chromophore (the tetrapyrrole molecule phycocyanobilin B) contained in spirulina are mainly responsible for its antioxidant activity. The phycocyanin chromophore is more effective at inhibiting oxidation than known natural antioxidants such as caffeic acid, zeaxanthin, and alpha-tocopherol [90]. Spirulina, as a high-protein product, is also a potential source of bioactive proteins and peptides. Several peptides derived from microalgae with in vitro or in vivo antioxidant activity have been described [91]. In insects, both low molecular weight antioxidants such as ascorbate, glutathione, and α-tocopherol and antioxidant enzymes (superoxide dismutase, catalase, ascorbate peroxidase, and glutathione transferase peroxidase) were found to be antioxidant agents [92]. Di Mattia et al. [93] found that the total phenolic content in grasshoppers is comparable to that found in fresh orange juice. Insects cannot synthesize polyphenolic compounds but can accumulate them based on their diet during the larval stage. Edible insects are a source of bioactive compounds that are typically derived from plants, including phenolic compounds, terpenoids, steroids, glycosides, organic acids, carotenoids, and sulfur compounds. Chitin from the exoskeleton of insects also exhibits important biological activity [94]. Some researchers have identified phenolic compounds in the cuticles and secretions from the defensive glands of insects belonging to the *Tenebrio molitor* family [95]. In the case of flavonoids, their presence in insects is associated with chemical defense, reproduction, and pigmentation [94]. Furthermore, Navarro del Hierro et al. [96] reported a significant positive correlation between total phenolic content (TPC) and antioxidant activity. Additionally, the possible antioxidant activity can increase after digesting insects, which yields numerous bioactive peptides. The antioxidant activity of edible insect protein hydrolysates and peptides is reported to be relatively high compared to other foods’ protein hydrolysates [97]. Most research on the bioactive properties of insects emphasizes their proteins, peptides, and amino acids [97].

### 3.7. Predicted Glycemic Index In Vitro

**Figure 2:**
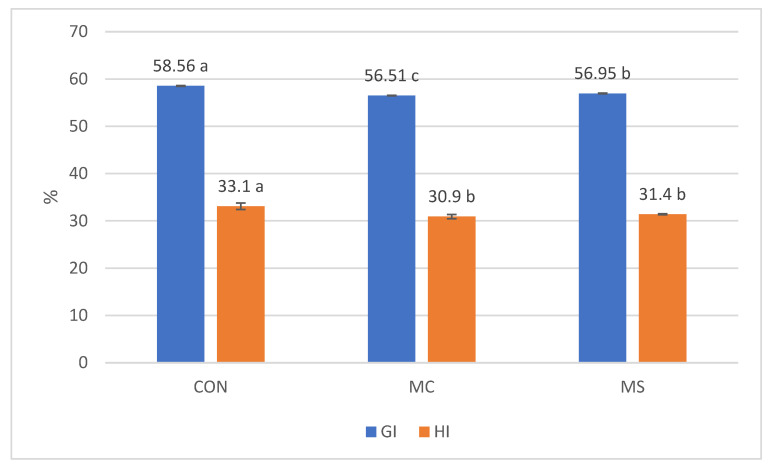
The in vitro starch hydrolysis index and in vitro predicted glycemic index values of muffins. HI—hydrolysis index of starch; GI—in vitro predicted glycemic index values; CON—control sample; MC—muffin with cricket; MS—muffin with spirulina. Data values of each parameter with different superscript letters are significantly different (*p* < 0.05); mean value (*n* = 3) ± SD.

The in vitro starch hydrolysis method is used to estimate the metabolic glycemic response to food products. The in vitro hydrolysis index of starch (HI) and in vitro predicted glycemic index values (GI) of muffins are shown in Figure 2. Carbohydrate-containing foods can be classified by their potential effects on postprandial blood glucose levels, which can be categorized as having a low, medium, or high in vitro predicted glycemic index (GI). All the muffins tested fall within the medium in vitro predicted glycemic index (GI) range of 55 to 70 [98]. However, we observe a significant decrease in the in vitro hydrolysis index of starch and the in vitro predicted glycemic index of the muffins that were supplemented—control muffin reached a value of 58.56 ± 0.06, muffin with cricket reached 56.51 ± 0.04, and muffin with spirulina reached 56.95 ± 0.07 (*p* < 0.05). We can conclude that using a higher level of supplementation with the studied powders would likely reduce the estimated GI to a low level—below 55. Modulating the postprandial blood glucose response by consuming low-glycemic index (low-GI) foods has beneficial health effects, warranting further research in this area. Various factors can help lower the glycemic index of starchy and heat-treated foods. The presence or inclusion of other food ingredients is crucial. Starch can interact with lipids and proteins through electrostatic and hydrophobic interactions. These interactions increase the organization of the starch structure and slow down its digestibility. They play a crucial role in modulating how easily starch can be digested, and this can occur under various conditions [99]. In turn, phenolic compounds can inhibit amylolytic enzymes, which delays glucose absorption and contributes to lower postprandial glycemia [100]. The enriched muffins had a higher polyphenol content than the control sample, which may indeed have affected enzyme activity. Another effect that may have been present in the muffins is the formation of networks of a viscous protein–fiber–starch that can trap the starch granules, reducing the availability of starch to digestive enzymes [101]. Due to the growing interest in low-glycemic-index products, the findings obtained are a crucial part of research into non-conventional protein sources. These sources, in addition to their nutritional benefits, probably can enhance the health-promoting properties of foods.

### 3.8. Consumer Acceptance Evaluation and Consumer Study

The senses of sight, taste, and smell play an essential role in selecting a particular food item. Therefore, especially when designing new products, organoleptic evaluation by potential consumers is critical. The results of the assessment of consumer acceptance of muffins, using a nine-point hedonic scale, are shown in Table 8. Overall, all the muffins received were acceptable to consumers and received high ratings. Consumers accustomed to the traditional color of muffins rated the control baked product highest for color (7.91 ± 1.24) (*p* < 0.05). Muffins containing spirulina, despite their distinct darker color, received high marks from respondents for this quality distinction (7.23 ± 1.52) (*p* < 0.05). The baked product enriched with cricket powder received the lowest rating in terms of color (6.60 ± 1.63) (*p* < 0.05). This variant also achieved the lowest score for smell (6.36 ± 2.04) (*p* < 0.05). Notably, the muffins with spirulina in terms of smell received a high score (7.79 ± 1.24), which was not significantly different (*p* < 0.05) from the smell of the control sample (7.19 ± 1.35). The intense green color and distinctive fishy taste and smell are identified as key problems in consumer acceptance of spirulina [26]. However, as indicated in this study, an appropriate level of supplementation, even for confectionery, can be acceptable and highly rated. Muffins enriched with microalgae also received the highest score for texture (8.07 ± 1.04), which may be related to their lowest hardness, gumminess, and chewiness recorded in the instrumental evaluation. According to consumers, the texture of the control muffins (7.41 ± 1.44) and those enriched with cricket powder (7.26 ± 1.59) were similar to one another (*p* < 0.05). It is worth noting that taste, a crucial factor in determining food quality, was rated similarly across all muffin types (ranging from 7.23 ± 1.60 to 7.73 ± 1.43). In the overall impression, similar scores were given to the control (7.59 ± 1.10) and spirulina-enriched (7.69 ± 1.21) baked goods, while significantly lower scores were given to cricket muffins by consumers (7.03 ± 1.38) (*p* < 0.05). Similarly, in the preference ranking method, muffins enriched with microalgae received the highest score (164 points), while muffins with insect powder received the lowest score (113 points). The samples tested were coded; however, before the study commenced, consumers were informed about the additives used to eliminate concerns about allergies. After receiving this information, reactions of disgust toward edible insects were evident. The color of spirulina is quite intense, and the muffins produced can be easily identified by their color (Figure 1). A specific aversion to eating insects may be a more significant predictor than neophobia itself when it comes to understanding the willingness to consume unconventional foods [36]. In our research, we aimed to conduct a consumer assessment of all characteristics, including color. However, in future studies, it is advisable to mask the color of the products to prevent suggesting the composition of the recipes being evaluated. This approach will help minimize any potential disgust factor during organoleptic evaluations.

Additionally, on the hedonic evaluation card, consumers were allowed to justify their rankings of the samples in terms of preference by listing the characteristics of the best and worst samples. The most frequently mentioned characteristics of the best sample that ranked first, which are muffins with spirulina, are taste (*n* = 27), color (*n* = 19), aroma (*n* = 10), and texture (*n* = 9). Two consumers mentioned such a characteristic as moisture. For the sample that ranked last, the cricket muffins, consumers were more specific in describing the worst features of the product. Respondents mentioned characteristics such as color (*n* = 4), texture (*n* = 4), taste (*n* = 3), smell (*n* = 3), unpleasant odor (*n* = 3), and strange smell (n = 2). Focusing on the negative aspects of insect-enriched muffins can be beneficial for enhancing the recipe. In a separate study on insect-enriched products, consumers noted a positive change in color, which is typically associated with whole grain products and perceived as healthier [59]. However, in our case with the muffins, the color achieved was not dark enough for such a comparison and appeared unattractive when compared to the lighter control sample. Color is a crucial feature of a product that can influence our choice even before we evaluate its taste. We can enhance it by masking the color, for example, by adding natural coloring substances like cocoa. We can similarly enhance scent by selecting natural aromas like vanilla, caramel, or citrus. In preparing the recipe, we intentionally opted for a very basic version to evaluate better the various characteristics that arise from the additives used.

In addition to the organoleptic evaluation, a consumer opinion test was conducted on two non-conventional protein sources: insect powder and spirulina. This test involved a series of statements that participants rated on a five-point Likert scale, ranging from “strongly disagree” to “strongly agree.” The consumer survey was carried out in two parts—before and after the organoleptic evaluation of the muffins—to compare the opinions gathered at both stages. Among consumers, 48.57% (*n* = 34) had never previously consumed products with a non-conventional protein source, while the remaining 51.43% (*n* = 36) had consumed such foods before. In general, consumers were eager to explore the taste of muffins (4.16) (Table 9). Moreover, females supported this statement more strongly (4.32) than males (3.83) (*p* < 0.05). Females were also more likely to indicate that all muffin variants look equally appetizing (3.83) than males (3.26) (*p* < 0.05). Consumers were positively surprised by the taste of the muffins (4.13) (Table 10). Furthermore, respondents indicated that they find muffins made with non-conventional protein sources acceptable and would be willing to eat them in the future (4.34). They also expressed interest in trying other baked goods that contain insect powder or spirulina (4.27). Consumers are confident in recommending baked goods made with these ingredients to others (4.16). These are essential findings, indicating that even European consumers, for whom insect- or microalgae-based foods are not a staple of their diet, can accept new products. This is also significant information for food producers (including confectionery and bakery products) looking to innovate their products. However, it should be emphasized that the consumers participating in the study were young people (aged 19–36) and came from an institution involved in food research. It is believed that young people in particular are more concerned about environmental changes and are therefore open to new food products containing non-conventional protein sources, including edible insects [102]. Additionally, as university students and employees, respondents may be better educated and more aware of the need to utilize non-conventional protein sources than non-students. Such experiences may also influence their openness and attitudes towards edible insects, microalgae, and foods containing them. Future research should also focus on older consumers, as generational differences strongly influence views on sustainable and healthy eating, and people from different generations have different eating behaviors [102].

There is a strong need to shift to a more sustainable diet that emphasizes resource efficiency, agrobiodiversity, and alternative food sources. A common trend is moving towards diets that are less reliant on animal products but high in protein. When developing new foods, especially those based on edible insects or microalgae, which are not typically consumed by Western populations, evaluating consumer acceptance is a crucial step [103]. Insects, as an alternative food source for humans, offer numerous health benefits, but there is often a consumer acceptance barrier to overcome. Many factors influence the acceptability of insect-based foods, including cultural norms; socio-demographic characteristics; social influences; psychological factors; environmental knowledge and awareness; as well as product characteristics such as appearance, visibility, and taste [104]. In general, processed insects (insect powder or protein concentrate powder) are more acceptable than whole insects; hence, this is the form best used for food fortification [104,105]. Iannuzzi et al. [106] studied consumer preferences for consuming insect- and spirulina-based foods, showing that rejection of products occurs due to neophobia when they are made entirely of unfamiliar ingredients. In contrast, associating new ingredients (such as insects or spirulina) with known foods reduced rejection. Regarding spirulina, its intense green color and distinctive taste and smell can pose challenges for consumer acceptance. The characteristic aroma of the microalgae is composed of numerous volatile organic compounds (VOCs), including hydrocarbons, ketones, aldehydes, esters, furans, and nitrogen-containing compounds, among others. Generally, they contribute a peculiar, oily, and seafood-like odor [107].

Despite the indicated problems with consumer acceptance, products containing non-conventional protein sources have gained popularity in recent years. Numerous studies have been conducted to document their effects on food products, including their sensory attributes. A consumer acceptance study conducted by Pauter et al. [59] on muffins with the addition of cricket powder showed that control muffins had the highest appearance scores compared to fortified baked goods. The addition of insect powder caused changes in overall appearance and color, which consumers rated as negative. However, the addition of the tested raw material caused an increase in the values of texture and flavor characteristics. Based on the overall rating results, it was suggested that a 2% replacement of wheat flour with cricket powder would be acceptable to consumers. On the other hand, in a study conducted on sweet and savory muffins with mealworm, buffalo worm, house cricket, and grasshopper [19], the control muffins received the highest marks in both sweet and savory versions. A comparison of the control muffins with samples containing *Acheta domesticus* showed that in the sweet variant, the addition of cricket powder significantly reduced the taste rating, although the other characteristics—appearance, aroma, texture, and overall acceptance—were not significantly different. In the savory variant, there were no significant sensory differences between the control sample and the one with the addition of *A. domesticus*. This means that house cricket can be a generally well-accepted ingredient in baked goods, especially in the salty version. In the case of spirulina muffins, research performed by Güroy [108] on muffins with fresh and powdered spirulina showed no significant change in the parameters of appearance, texture, taste, and acceptability. However, significant changes were noted in the characteristics of odor and color. The highest score in terms of odor was achieved by muffins with 4‰ fresh spirulina, and in terms of color by samples with 6‰ and 8‰ fresh spirulina. On the other hand, a study conducted on cookies with the addition of microalgae such as *A. platensis* and *C. vulgaris* [109] concluded that there was less preference for cookies with added *C. vulgaris* compared to spirulina. In terms of color, the preferred cookies contained 2% *Chlorella*, while in terms of flavor, tasters preferred products with *A. platensis*. Regarding texture, there were no significant differences between spirulina (2% and 6%) and *C. vulgaris* (2%), while *C. vulgaris* 6% was less appreciated. In flavor and global appreciation, the preferred cookie was *A. platensis* 2%, while *A. platensis* 6% and *C. vulgaris* 2% had similar results. Moreover, a study conducted on vegan muffins with *C. vulgaris* [57] found no significant differences in sensory evaluation parameters despite differences in microstructure and texture.

## 4. Research Limitations and Safety Concerns

This study has certain limitations. One of them is that the consumer panel participating in the consumer evaluation of fortified products consisted mainly of young people (aged 19–36) associated with a university. Their education and university-acquired knowledge of nutrition may have influenced the evaluation results. Further research should expand the study group to include older people and those not involved in nutrition.

Although the FDA has granted spirulina GRAS status, its use still has several limitations, and in Western cultures, spirulina-supplemented products are not yet widely available on store shelves. However, as demonstrated in the study presented, foods containing spirulina have an advantage over other products enriched with non-conventional additives, such as edible insects, and are more likely to be accepted by consumers. This is probably because spirulina is perceived as a plant-based product. Another limitation to the broader use of spirulina is consumers’ poor knowledge of its rich composition and potential health and environmental benefits [26]. While edible insects are not a major part of the European diet, the European Commission Implementing Regulation (EU) 2017/2470, enacted on 20 December 2017, established a new food register that includes insects. As of now, four species of insects are approved for consumption in the European Union: yellow mealworm (*Tenebrio molitor*), migratory locust (*Locusta migratoria*), house cricket (*Acheta domestica*), and lesser mealworm (*Alphitobius diaperinus*) [110]. The new regulations for the novel food list oversee food safety and the quality of products derived from insects. Edible insects are viewed as a safe alternative in animal production, offering an innovative solution. However, it is crucial to implement processing procedures and adhere to sanitary guidelines to reduce the risk of foodborne pathogen contamination [111].

Another risk associated with consuming insect-derived foods is the potential for allergic reactions. Similar to other protein sources, such as milk, shellfish, or soy, eating insects can trigger allergies in susceptible individuals. Symptoms of these allergic reactions may include rashes, nausea, diarrhea, eczema, and in severe cases, anaphylactic shock [112]. Adverse reactions that occur after consuming insects may be linked to cross-reactivity with other food allergens related to their taxonomy, such as shellfish. Additionally, there is a possibility of cross-reactivity with inhalant allergens, like house dust mites (HDMs). This connection stems from the presence of common allergens found among invertebrates, including tropomyosin (TM), arginine kinase (AK), and glycerol-3-phosphate aldehyde dehydrogenase [113].

## 5. Conclusions

Edible insects and microalgae have recently gained significance as food ingredients due to their exceptional nutritional and predicted health-promoting benefits as well as their suitability for sustainable large-scale food production. The current study assessed the impact of non-conventional protein sources, including house cricket and spirulina powders, on the quality of popular confectionery products. The enriched muffins were primarily distinguished by their higher content of high-quality protein. Additionally, they contained higher levels of selected minerals. The in vitro antioxidant capacity against ABTS·+ and DPPH·, as well as TPC, increased in the fortified products. Additionally, using non-conventional additives resulted in a lower in vitro predicted glycemic index. Despite the altered physical properties, including a much darker color, muffins with spirulina added in the ranking method received the highest number of points awarded by consumers. Importantly, consumers did not notice any significant difference in taste between the control muffins and those enriched with edible insects or spirulina.

The results of this study highlight the many advantages of incorporating edible insects and microalgae into traditional foods. Although these ingredients are still regarded as unusual foods in European culture, increasing consumer interest in health-promoting products with clean labels suggests a promising future for edible insects and microalgae as new food ingredients. By carefully selecting suitable amounts of these non-conventional additives, it is possible to create high-quality, nutritious products that are not only acceptable to consumers but also rated higher than traditional alternatives. Therefore, the results from the consumer assessment in this study are promising for the future and motivate food producers to incorporate edible insects or microalgae into a broader range of traditional products.

## Figures and Tables

**Table 1:** Color determinants of muffins.

Sample	L*	a*	b*	C*	h°	ΔE	WI
CON	71.19 ± 0.99 ^a^	2.80 ± 0.32 ^c^	25.68 ± 0.74 ^b^	25.73 ± 0.68 ^b^	83.80 ± 0.61 ^a^	-	61.31
MC	65.08 ± 0.81 ^b^	3.41 ± 0.25 ^b^	20.13 ± 0.42 ^c^	20.33 ± 0.47 ^c^	80.37 ± 0.66 ^b^	8.28	59.55
MS	32.62 ± 1.06 ^c^	7.78 ± 0.71 ^a^	30.43 ± 0.71 ^a^	31.41 ± 0.60 ^a^	75.72 ± 0.88 ^c^	39.17	25.67

Lightness (L*) and color (a*—redness, b*—yellowness). Chroma (C*) and hue (h°). ΔE—total color difference. WI—whiteness index. CON—control sample; MC—muffin with cricket; MS—muffin with spirulina. In each column, values marked with the same letters do not differ significantly at *p* < 0.05 (Tukey’s post hoc test); mean value (*n* = 3) ± SD.

**Table 2:** Textural properties of muffins.

Sample	Hardness (g)	Cohesiveness	Springiness (mm)	Gumminess (g)	Chewiness (mJ)
CON	2754.33 ± 90.64 ^a^	0.51 ± 0.01 ^b^	10.37 ± 0.58 ^a^	1416.00 ± 21.00 ^a^	144.03 ± 10.01 ^a^
MC	1877.50 ± 76.36 ^b^	0.56 ± 0.02 ^a^	9.98 ± 0.50 ^a^	1049.00 ± 23.71 ^b^	102.75 ± 6.08 ^b^
MS	1434.00 ± 80.73 ^c^	0.56 ± 0.01 ^a^	9.83 ± 0.71 ^a^	861.00 ± 49.52 ^c^	77.20 ± 2.63 ^c^

CON—control sample; MC—muffin with cricket; MS—muffin with spirulina. In each column, values marked with the same letters do not differ significantly at *p* < 0.05 (Tukey’s post hoc test); mean value (*n* = 3) ± SD.

**Table 3:** Nutritional value of muffins and powders used.

Sample	Protein	Fat	Carbohydrates	Ash	Moisture	Energy Value
	g/100 g FM					kcal/100 g
CON	6.79 ± 0.05 ^b^	14.66 ± 0.08 ^b^	40.22 ± 0.4 ^a^	1.19 ± 0.01 ^b^	37.14 ± 0.53 ^a^	319.98
MC	7.19 ± 0.06 ^a^	15.54 ± 0.11 ^a^	38.47 ± 0.77 ^b^	1.21 ± 0.02 ^b^	37.59 ± 0.45 ^a^	322.50
MS	7.10 ± 0.05 ^a^	14.59 ± 0.09 ^b^	39.91 ± 0.4 ^a^	1.28 ± 0.04 ^a^	37.12 ± 0.38 ^a^	319.35
Cricket powder	73.24 ± 0.17	12.01 ± 0.04	0.8 ± 0.08	4.43 ± 0.03	9.52 ± 0.18	404.25
Spirulina powder	70.20 ± 0.23	5.60 ± 0.01	11.93 ± 0.12	6.21 ± 0.03	6.06 ± 0.29	378.92

CON—control sample; MC—muffin with cricket; MS—muffin with spirulina. In each column, values marked with the same letters do not differ significantly at *p* < 0.05 (Tukey’s post hoc test); mean value (*n* = 3) ± SD.

**Table 4:** Amino acid content of muffins and used powders (mg/g protein).

Amino Acids	Muffins			Cricket Powder	Spirulina Powder	WHO/FAO/UNU ^1^	
	CON	MC	MS			(mg/g Protein)	(mg/kg Body Mass/Day)
Isoleucine *	3.74 ± 0.07 ^b^	3.77 ± 0.06 ^b^	4.03 ± 0.05 ^a^	47.8 ± 0.2	37.5 ± 0.09	30	20
Leucine *	7.42 ± 0.1 ^b^	7.45 ± 0.08 ^b^	7.93 ± 0.1 ^a^	56.1 ± 0.12	63.7 ± 0.12	59	39
Lysine *	3.56 ± 0.09 ^b^	4.10 ± 0.05 ^a^	4.16 ± 0.11 ^a^	39.3 ± 0.15	34.6 ± 0.08	45	30
Methionine *	4.0 ± 0.04 ^c^	4.39 ± 0.04 ^b^	4.59 ± 0.08 ^a^	15.5 ± 0.07	23.2 ± 0.07	16	10
Cysteine *	2.8 ± 0.05 ^b^	3.09 ± 0.04 ^a^	3.16 ± 0.06 ^a^	10.2 ± 0.07	10.5 ± 0.02	6	4
Total sulphur a.a. **	**6.8**	**7.48**	**7.75**	**25.7**	**33.7**	**22**	**14**
Phenylalanine *	4.75 ± 0.11 ^b^	4.77 ± 0.07 ^ab^	4.98 ± 0.09 ^a^	23.9 ± 0.05	33.0 ± 0.07	30	25
Tyrosine	2.63 ± 0.08 ^b^	2.81 ± 0.1 ^ab^	2.88 ± 0.11 ^a^	35.0 ± 0.05	30.7 ± 0.09	32	
Total aromatic a.a. ***	**7.38**	**7.58**	**7.86**	**58.9**	**63.7**	**30**	**25**
Threonine *	3.17 ± 0.07 ^b^	3.41 ± 0.12 ^ab^	3.59 ± 0.1 ^a^	27.0 ± 0.12	37.5 ± 0.11	23	15
Valine *	4.53 ± 0.1 ^b^	4.92 ± 0.1 ^a^	4.93 ± 0.14 ^a^	39.2 ± 0.15	42.5 ± 0.12	39	26
**Total essential a.a.**	**36.6**	**38.71**	**40.25**	**294**	**313.2**	**242**	**165**
Histidine	2.11 ± 0.05 ^b^	2.39 ± 0.05 ^a^	2.32 ± 0.07 ^a^	20.5 ± 0.07	14.5 ± 0.08		
**No essential a.a.**							
Aspartic acid	6.63 ± 0.14 ^b^	6.89 ± 0.11 ^b^	7.22 ± 0.1 ^a^	66.4 ± 0.2	72.0 ± 0.15		
Serine	4.71 ± 0.09 ^b^	4.92 ± 0.12 ^ab^	5.02 ± 0.07 ^a^	34.6 ± 0.07	35.1 ± 0.12		
Glutamic acid	26.1 ± 0.18 ^a^	25.7 ± 0.17 ^a^	26.2 ± 0.2 ^a^	88.3 ± 0.25	109.6 ± 0.22		
Proline	9.03 ± 0.12 ^a^	8.98 ± 0.09 ^a^	9.04 ± 0.11 ^a^	63.6 ± 0.2	29.2 ± 0.1		
Glycine	2.98 ± 0.09 ^b^	3.3 ± 0.06 ^a^	3.38 ± 0.07 ^a^	35.9 ± 0.07	35.8 ± 0.1		
Alanine	3.59 ± 0.1 ^b^	4.56 ± 0.12 ^a^	4.47 ± 0.1 ^a^	63.4 ± 0.2	56.0 ± 0.2		
Arginine	3.43 ± 0.1 ^b^	4.1 ± 0.09 ^a^	4.02 ± 0.08 ^a^	44.6 ± 0.15	44.0 ± 0.17		
**Total a.a.**	**95.18**	**98.55**	**101.92**	**711.3**	**709.4**		

* Essential amino acids, ** Methionine + cysteine, *** Phenylalanine + tyrosine, a.a. = amino acids, ^1^ (WHO. 2007 [73]). CON—control sample; MC—muffin with cricket; MS—muffin with spirulina. In each row, values marked with the same letters do not differ significantly at *p* < 0.05 (Tukey’s post hoc test); mean value (*n* = 3) ± SD.

**Table 5:** Chemical score of protein quality (CS) of muffins and powders used.

Amino Acids	Chemical Score of Protein Quality (CS) (%)				
	CON	MC	MS	Cricket Powder	Spirulina Powder
Isoleucine	12.47	12.57	13.43	159.33	125.00
Methionine	25.00	27.44	28.69	96.88	145.00
Leucine	12.58	12.63	13.44	95.08	107.97
Threonine	13.78	14.83	15.61	117.39	163.04
Lysine	7.91	9.11	9.24	87.33	76.89
Phenylalanine + Tyrosine	11.9	12.2	12.67	95	102.74
Valine	11.62	12.64	12.64	100.51	108.97
EAAI	17.12	18.38	19.04	112.84	123.09
Amino acid limiting	Lysine	Lysine	Lysine	Lysine	Lysine

CON—control sample; MC—muffin with cricket; MS—muffin with spirulina.

**Table 6:** Mineral content of muffins and powders used.

Sample	Ca	Mg	K	Na	Fe	Zn
	mg/100 g FM					
CON	43.44 ± 0.77 ^b^	11.50 ± 0.41 ^b^	108.76 ± 2.15 ^b^	185.20 ± 1.59 ^b^	0.75 ± 0.03 ^b^	0.51 ± 0.04 ^b^
MC	48.05 ± 0.15 ^a^	11.98 ± 0.42 ^b^	126.65 ± 0.34 ^a^	190.86 ± 2.20 ^b^	0.86 ± 0.05 ^b^	0.78 ± 0.02 ^a^
MS	48.83 ± 0.29 ^a^	16.30 ± 0.05 ^a^	126.72 ± 0.32 ^a^	251.17 ± 0.81 ^a^	1.50 ± 0.05 ^a^	0.60 ± 0.05 ^b^
Cricket powder	87.18 ± 0.37	114.80 ± 0.78	1296.22 ± 3.03	408.26 ± 2.99	4.42 ± 0.07	22.65 ± 0.65
Spirulina powder	99.33 ± 1.29	245.42 ± 1.55	1448.41 ± 1.21	606.93 ± 0.28	35.59 ± 0.20	1.62 ± 0.06

CON—control sample; MC—muffin with cricket; MS—muffin with spirulina. In each column, values marked with the same letters do not differ significantly at *p* < 0.05 (Tukey’s post hoc test); mean value (*n* = 3) ± SD.

**Table 7:** Antioxidant properties of muffins.

Sample	TPC (mg GAE/100 g)	DPPH· (mM TE/100 g)	ABTS·+ (mM TE/100 g)
CON	18.42 ± 0.06 ^c^	0.018 ± 0.002 ^c^	0.126 ± 0.001 ^c^
MC	26.47 ± 0.27 ^a^	0.087 ± 0.003 ^a^	0.211 ± 0.005 ^a^
MS	20.20 ± 0.35 ^b^	0.053 ± 0.002 ^b^	0.163 ± 0.004 ^b^

CON—control sample; MC—muffin with cricket; MS—muffin with spirulina. In each column, values marked with the same letters do not differ significantly at *p* < 0.05 (Tukey’s post hoc test); mean value (*n* = 3) ± SD.

**Table 8:** Consumer acceptance analysis and results of preference ranking of muffins.

Sample	Color	Smell	Texture	Taste	Overall Impression	Ranking
CON	7.91 ± 1.24 ^a^	7.19 ± 1.35 ^a^	7.41 ± 1.44 ^b^	7.50 ± 1.19 ^a^	7.59 ± 1.10 ^a^	143
MC	6.60 ± 1.63 ^c^	6.36 ± 2.04 ^b^	7.26 ± 1.59 ^b^	7.23 ± 1.60 ^a^	7.03 ± 1.38 ^b^	113
MS	7.23 ± 1.52 ^b^	7.79 ± 1.24 ^a^	8.07 ± 1.04 ^a^	7.73 ± 1.43 ^a^	7.69 ± 1.21 ^a^	164

CON—control sample; MC—muffin with cricket; MS—muffin with spirulina. In each column, values marked with the same letters do not differ significantly at *p* < 0.05 (Tukey’s post hoc test); mean value (*n* = 70) ± SD.

**Table 9:** Results of statements before the sensory evaluation of the muffins (1 = strongly disagree; 5 = strongly agree) on the total sample and by gender.

Statement	Mean ± SD	DF	*p*-Value	Male Mean ± SD	Female Mean ± SD	F-Value
I know the benefits of consuming non-conventional protein sources (e.g., insects, microalgae)	3.60 ± 1.18	1	0.966	3.61 ± 1.16	3.60 ± 1.21	0.0018
I am curious about the taste of muffins that have had spirulina and insect powder added	4.16 ± 0.99	1	0.049 *	3.83 ± 1.40	4.32 ± 0.66	4.019
All muffin variants look equally appetizing	3.64 ± 1.08	1	0.036 *	3.26 ± 1.18	3.83 ± 0.99	4.527
The non-conventional additives made me reluctant to try the muffins	2.37 ± 1.28	1	0.138	2.70 ± 1.33	2.21 ± 1.23	2.252

SD—standard deviation; DF—Degree of Freedom; Signif. Codes: * *p* < 0.05, Tukey test; mean value (*n* = 70) ± SD.

**Table 10:** Results of statements after the sensory evaluation of the muffins (1 = strongly disagree; 5 = strongly agree) on the total sample and by gender.

Statement	Mean ± SD	DF	*p*-Value	Male Mean ± SD	Female Mean ± SD	F-Value
The muffins with non-conventional additives positively surprised me in terms of taste	4.13 ± 1.03	1	0.334	3.96 ± 1.19	4.21 ± 0.95	0.947
Muffins with non-conventional protein sources are acceptable to me, and I would eat them in the future	4.34 ± 0.88	1	0.974	4.35 ± 1.07	4.34 ± 0.79	0.001
I would be interested in trying other baked goods with insect powder or spirulina	4.27 ± 0.93	1	0.543	4.17 ± 1.15	4.32 ± 0.81	0.372
I would recommend trying baked goods made with insect powder or spirulina to others	4.16 ± 0.86	1	0.637	4.09 ± 1.00	4.19 ± 0.80	0.255

SD—standard deviation; DF—Degree of Freedom; Signif. Codes: *p* < 0.05, Tukey test; mean value (*n* = 70) ± SD.

## Data Availability

The original contributions presented in the study are included in the article; further inquiries can be directed to the corresponding author.
